# Self-injury, suicide ideation, and sexual orientation: differences in causes and correlates among high school students

**DOI:** 10.5249/jivr.v8i1.545

**Published:** 2016-01

**Authors:** Whitney DeCamp, Nicholas W.Bakken

**Affiliations:** ^*a*^Associate Professor of Sociology, Western Michigan University, Michigan,USA.; ^*b*^Assistant Professor of Sociology, University of Wisconsin-La Crosse,Wisconsin, USA.

**Keywords:** Non-suicidal self-injury, Suicide ideation, Sexual minority youth

## Abstract

**Background:**

Research has suggested that sexual minority youth are more likely to experience a number of behavioral and health-related risk factors due to their exposure to negative attitudes and beliefs about sexual minorities. Few studies, however, have examined the prevalence of non-suicidal self-injury (NSSI) among sexual minority youth. With self-cutting and suicidal ideation common in middle and high schools, understanding the antecedents and correlates of such behavior may help identify troubled students and initiate preventative measures.

**Methods:**

Bivariate probit regression analyses are performed using data from 7,326 high school students collected via the Delaware Youth Risk Behavior Survey.

**Results:**

Results indicate that bullying victimization, fighting, substance use, sexual behavior, depression, and unhealthy dieting behaviors were generally associated with NSSI and suicidal ideation. Some effects - including those from sexual activity, substance use, and unhealthy dieting behaviors significantly differed based on gender and orientation.

**Conclusions:**

Risk factors for suicide and NSSI vary by gender and orientation. Both prevention/intervention specialists and researchers should consider the intersection of these risk factors with sexual orientation in their efforts.

## Introduction

It is well known that adolescents face a variety of challenges as they make the developmental transition from high school into young adulthood. Youth are more likely to engage in antisocial behaviors and experience adverse life-threatening conditions, such as being bullied by their peers, substance abuse and other health risk behaviors.^1^ Accumulating evidence suggests that these issues and challenges are further exacerbated for sexual minority (i.e., gay, lesbian, bisexual, and questioning) youth. Sexual minority youth have been found to be more likely than heterosexual youth to engage in non-suicidal self-injury (NSSI), experience depressive symptoms, abuse substances, experience suicidal ideation (Suicidal ideation refers to thinking about, considering, or planning for suicide),^i^ and attempt suicide.^3-8^ Understanding that these self-harming behaviors often originate during adolescence and may transcend into adulthood,^9^ it is important for researchers and clinicians to recognize and identify the risk factors associated with NSSI and suicidal ideation during early adolescent development to aid in effective intervention and treatment strategies targeted towards sexual minority youth.

Sexual minority youth are often faced with dealing with an array of stressors relating to having a stigmatized identity.^10^ Despite the fact that public attitudes have become more socially accepting in recent years, the prescribed gender roles for adolescents remain pervasive.^11,12^ Sexual minority youth therefore endure numerous negative experiences, including social rejection and diminished peer and familial support, discrimination, and verbal and physical abuse.^4,13,14^ Several recent studies have demonstrated noticeably more health risk behaviors and depreciative health outcomes for sexual minority youth compared to their heterosexual peers.^6,15,16,17^ For instance, results from the Youth Risk Behavior Surveillance System (YRBS) conducted from 2001 to 2009^ii^ showed that 39% of sexual minority youth indicated feeling sad or hopeless in the past 2 weeks compared to 29% of heterosexual youth.^18^ Those data also indicated that sexual minority youth were more than twice as likely as heterosexual youth to have considered attempting suicide in the past year (30% vs. 12%). Furthermore, studies have also indicated a strong link between sexual minority youth and suicide attempts.^19,20,21^

A much smaller body of literature also suggests that sexual minority youth are more likely to engage in non-suicidal self-injury (NSSI).^22,23,24^ NSSI is most often referred to as the direct, deliberate destruction or alteration of body tissue devoid of any conscious suicidal intent,^25,26^ and includes the intentional cutting, scraping or burning on ones body. One study of 2,548 high school students found that youth (particularly females) that were self-identified as a sexual minority were more likely to self-injure (18% vs. 5%) compared to heterosexual students.^27^ A similar study of 1,032 adolescents ranging in ages from 13 to 19 in Boston found that both male (42% vs. 3%) and female (14% vs. 7%) sexual minorities were more likely to engage in self-harming behaviors when compared to their heterosexual counterparts.^4^ It has been suggested that sexual minority adolescents may use NSSI as a coping strategy as a way to deal with their stigmatized identities, low self-esteem and depression,^3^ however little is known regarding the actual prevalence of this behavior among this population. 

In addition to depression, suicidal ideation, and self-harming behaviors, sexual minority youth are highly vulnerable to peer victimization, substance abuse and health-related risk behaviors, such as eating disorders.^19,23,28,29^ While bullying is a pervasive problem for all adolescents, it is particularly concerning for sexual minorities due to increased victimization rates. ^30^ Reports from the Washington D.C. YRBS indicated that 31% of sexual minority youth reported being bullied in the past year compared to 17% of heterosexual youth and that these students were more likely to skip school because they felt unsafe.^31^ These results suggest that the social sphere in which sexual minority youth find themselves is often an antagonizing one. 

Empirical research has also consistently confirmed higher rates of substance use and eating disorders among sexual minority youth.^15,32^ One meta-analysis found that sexual minority youth are almost three times more likely than heterosexual youth to use illicit substances.^5^ Similarly, a study of 4,159 high school students found that having a sexual minority orientation was associated with an increased lifetime frequency of use of cocaine, anabolic steroids, inhalants, and other illegal and injectable drugs.^15^ Similar evidence regarding the presence of increased eating disorders among sexual minority youth has also been discovered. A recent study found that compared to heterosexual youth, sexual minority youth had higher rates of binge eating and purging. ^33^ Finally, results from the 2004 Minnesota Student Survey found that sexual minority males were more likely (53%) to report any of 5 disordered eating behaviors (fasting, smoking to control weight, using diet pills or laxatives, vomiting, and binge eating) when compared to heterosexual males (39%). ^34^ Given the behavioral and health risks incurred among sexual minority youth, this study aims to examine and compare the specific risk factors associated with male and female sexual minority and non-sexual minority youth who engage in NSSI and suicidal ideation.

i. Suicidal ideation refers to thinking about, considering, or planning for suicide.^2^

ii.These data come from nine different sites that assessed sexual identity data between the years of 2001 and 2009. The percentages indicated represent the median percentage across all nine sites for both sexual minority and heterosexual youth.

## Methods

**Data Collection and Sample**

The data used in this study come from the 2005, 2007 and 2009 Delaware High School Youth Risk Behavior Survey (YRBS-H).^iii^ The survey includes approximately 130 questions developed by the Centers for Disease Control and Prevention and the University of Delaware Center for Drug and Alcohol Studies to estimate prevalence rates for risk behaviors. The survey is administered biannually to a random sample of ninth through twelfth grade classrooms in Delaware public and public-charter schools.^iv^ Students are asked to complete a self-administered, anonymous, questionnaire. Eighty-four percent of students enrolled in the randomly selected classrooms were present on their classroom's days of administration, 98% (n=7,781) of whom agreed to participate and returned a completed survey.Three years of data (spanning five calendar years) were merged for this study to provide an adequate sample size for sexual minority youth. Due to the already limited sample size for these groups, every effort was made to avoid losing cases due to missing data; multiple imputation was used to address missing data for non-dependent variables. Six percent of cases were ultimately deleted from the data, as the participants did not provide a response for the dependent variables used for this study and/or for the sexual orientation question, resulting in a final sample of 7,326 high school students. The participants were 48.2% (n=3534) heterosexual males, 44.9% (n=3291) heterosexual females, 4.6% (n=340) sexual minority females, and 2.2% (n=161) sexual minority males.^v^

**Variables**

The primary dependent variable for this study is the question, "During the past 12 months, did you do something to purposely hurt yourself without wanting to die, such as cutting, scraping, or burning yourself on purpose?" Thirteen percent of high school students in the sample reported engaging in NSSI during the past year, which falls around the average prevalence rates found in previous high school samples.^35,36^ The prevalence of NSSI varies dramatically by gender and orientation, with seven percent of heterosexual males, fourteen percent of heterosexual females, thirty-three percent of sexual minority males, and forty-eight percent of sexual minority females engaging in NSSI. An additional dependent variable of interest is, "During the past 12 months, did you ever seriously consider attempting suicide?" As with NSSI, the prevalence of considering suicide varies by gender and orientation, with eight percent of heterosexual males, thirteen percent of heterosexual females, thirty-six percent of sexual minority males, and forty-two percent of sexual minority females having considered suicide. Both variables provided dichotomous yes/no response categories. As will be discussed in greater detail in the analysis section, these variables are moderately correlated (r = .44) and will be analyzed as related outcomes. The descriptive statistics for these and all other variables to be used are presented in Table 1.

**Table 1:** Descriptive statistics.

	Mean/Percent	SD	Min	Max	Factor Loading
**Dependent Variables**					
Cutting	13%	-	-	-	-
Considered Suicide	13%	-	-	-	-
**Independent Variables**					
Sad/Hopeless	27%	-	-	-	-
Victim of Sexual Assault	9%	-	-	-	-
Fasting	11%	-	-	-	-
Diet pills	4%	-	-	-	-
Purging	4%	-	-	-	-
**Bullied Factor**					
Harassed	1.27	.84	1.00	5.00	.73
Assaulted	1.24	.79	1.00	5.00	.78
Threatened	1.18	.90	1.00	8.00	.74
Stolen/damaged property	1.41	1.07	1.00	8.00	.79
**Fighting Factor**					
In a fight	1.69	1.43	1.00	8.00	.80
Injured in a fight	1.08	.43	1.00	5.00	.80
Fought at school	1.21	.82	1.00	8.00	.87
**Substance Use**					
Cigarettes	1.74	1.63	1.00	7.00	.83
Alcohol	1.89	1.30	1.00	7.00	.81
Marijuana	1.65	1.34	1.00	6.00	.81
**Hardcore Drug Use**					
Cocaine	1.09	.56	1.00	6.00	.93
Inhalants	1.10	.55	1.00	6.00	.93
**Sexual Behavior**					
Lifetime number of partners	1.73	2.07	.00	6.00	.94
Recent number of partners	1.27	1.48	.00	6.00	.94

Most independent variables used here are formed through factor extraction as a part of factor analysis. These factors include having been bullied, being in fights, substance use, hardcore drug use, and sexual behavior.

**Bullied: **The bullying factor was constructed using the following four variables: "During the past 30 days, on how many days were you harassed or bullied on school property?", "During the past 30 days, on how many days has someone tried to hurt you by hitting, punching, or kicking you on school property?", "During the past 12 months, how many times has someone threatened or injured you with a weapon such as a gun, knife, or club on school property?," and "During the past 12 months, how many times has someone stolen or deliberately damaged your property, such as your car, clothing, or books on school property?" The first two of these questions included possible responses of 0 days, 1 day, 2 or 3 days, 4 or 5 days, and 6 or more days. The latter questions included responses of 0 times, 1 time, 2 or 3 times, 4 or 5 times, 6 or 7 times, 8 or 9 times, 10 or 11 times, and 12 or more times. When combined together with factor extractions, the factor accounts for 58% (2.30 Eigenvalues) of the variance (alpha = .75). As with all other factors created for these analyses, this factor is standardized to have a mean of zero and a standard deviation of one.

**Fighting: **The fighting factor was also constructed using three indicators. These questions include: "During the past 12 months, how many times were you in a physical fight?", "During the past 12 months, how many times were you in a physical fight in which you were injured and had to be treated by a doctor or nurse?", and "During the past 12 months, how many times were you in a physical fight on school property?" These questions use the same number of times format as used for the final bullying measure (0 times to 12 times or more), except for the injury question which concludes with 6 or more times. The factor extracted from these questions accounts for 68% (2.03 Eigenvalues) of the combined variance (alpha = .65).

**Substance Use: **As before, three indicators were used to capture this factor, including: "During the past 30 days, on how many days did you smoke cigarettes?", "During the past 30 days, on how many days did you have at least one drink of alcohol?", and "During the past 30 days, how many times did you use marijuana?" For cigarettes and alcohol, possible responses included 0 days, 1 or 2 days, 3 to 5 days, 6 to 9 days, 10 to 19 days, 20 to 29 days, and all 30 days. Marijuana use, however, included responses of 0 times, 1 or 2 times, 3 to 9 times, 10 to 19 times, 20 to 39 times, and 40 or more times. The factor extracted from these variables accounts for 66% (1.99 Eigenvalues) of the variance (alpha = .74).

**Hardcore Drug Use: **This factor, as with the ones to follow, was constructed using two indicators. Using only two indicators in the construction of a factor limits the advantages of factor extraction, as both variables will always load equally on the factor due to the nature of calculating factor loadings. Essentially, factor extraction in this situation is statistically equivalent to forming an index. For the analyses in this study, however, factor extractions were still used on these item groupings, as this keeps the constructs more uniform in design and result in constructs consistently being standardized to the same metric. This particular factor was created using the questions, "During the past 30 days, how many times did you use any form of cocaine, including powder, crack, or freebase?" and "During the past 30 days, how many times have you sniffed glue, breathed the contents of aerosol spray cans, or inhaled any paints or sprays to get high?" Both had the response category also used for marijuana use (0 times to 40 or more times). These variables scaled together with moderate reliability (alpha = .85).

**Sexual Behaviors: **Two questions were used to measure sexual activity: "During your life, with how many people have you had sexual intercourse?" and "During the past 3 months, with how many people did you have sexual intercourse?" Both questions provide responses for each possible number of people, concluding with 6 or more people. These variables scale together moderately well (alpha = .85).

**Additional Independent Variables: **In addition to the factors being used as predictors of cutting and considering suicide, several single indicator items will also be included, all of which are dichotomous yes/no questions. First, a measure will be used to tap into depression: "During the past 12 months, did you ever feel so sad or hopeless almost every day for two weeks or more in a row that you stopped doing some usual activities?" Though this single question is not a true measure of depression, which is a more complex concept and would require a large battery of questions, this commonly used indicator taps a symptom of depression and can be considered a proxy for depression. Second, a question about sexual assault: "Have you ever been physically forced to have sexual intercourse when you did not want to?" Third, a series of three questions will be used to measure unhealthy weight loss strategies: "During the past 30 days, did you go without eating for 24 hours or more (also called fasting) to lose weight or to keep from gaining weight?", "During the past 30 days, did you take any diet pills, powders, or liquids without a doctor's advice to lose weight or to keep from gaining weight? (Do not include meal replacement products, such as Slim Fast)", and "During the past 30 days, did you vomit or take laxatives to lose weight or to keep from gaining weight?" These individual indicators that serve as proxies for measures of eating disorders were not combined into a single factor because they correspond with different types of eating abnormalities and weight management, and therefore may have differing effects.

**Analysis**

Typically, a logit-based regression (i.e. logistic regression) is used with dichotomous dependent variables because it accounts for the non-continuous nature of such variables by using the natural log rather than the variable itself. In this case, however, an added characteristic in the data, the correlated dependent variables, provides an additional need in the choice of a regression technique. Though the estimates of separately performed logistic regressions would indeed be accurate in describing the relationships between the independent variables and each of the dependent variables, using a technique that recognizes and controls for the correlation between the related dependent variables provides the added benefit of allowing for each dependent variable to control for each other without actually having to enter the alternative variable itself into the model as a cause of the other.^37^ Therefore, the models in this study will be estimated using bivariate probit regression. For purposes of interpreting the output of such estimations, the substantive conclusions of a probit model is virtually always the same as a logit model for most models.^38^ Moreover, the coefficients can be interpreted similar to those of a logistic regression with one difference. In a logistic regression, the dependent variable is the log of the odds that some characteristic is present, whereas a probit model estimates the cumulative normal probability that the characteristic is present.^38^ In other words, the estimated value of the dependent variable in a probit model is analogous to a Z score of the probability that Y= 1.

In order to address both gender and sexual orientation together, the models will be analyzed once for each of the following four groups: heterosexual males, sexual minority males, heterosexual females, and sexual minority females. Because the emphasis in this study is on sexual orientation, results will largely focus on the differences between sexual orientations within gender category. Due to the large differences in sample sizes between heterosexual and sexual minority youth in these data, there are consequentially power differences in the samples. As a result (and in addition to other factors), differences in significance do not necessarily mean a significant difference between groups. To properly test for differences, the differences of coefficient test will be used to determine significant changes between groups.^39^

iii. The survey methodology was the same for each year, though some survey questions not used in the present study were added and/or dropped in different years.

iv. There are, as in any state, a number of schools that do not fit this category, including secular and religious private schools, alternative schools, detention schools, and specialized school-replacement programs (e.g., programs for pregnant students). Though these schools serve a minority of high school students, it is an unfortunate limitation these smaller populations could not be included in these data. Because the majority of high school students attend traditional public schools, it is unlikely that this will impact the generalizability of this study’s findings.

v. Participants were given the following options in response to being asked which “best describes” them: Heterosexual (straight), Gay or lesbian, Bisexual, or Not sure. Due to limited sample sizes in the latter three groups, each are combined for this study into a single “sexual minority” category. We do not contend that these youth could be entirely described as homosexual or gay. Rather, this category contains a range of youth who have the common trait of not describing themselves as heterosexual.

## Results

The results for the bivariate probit regressions for males are presented in Table 2^vi^. Beginning at the top, being the victim of bullying is significantly related to both self-injury and suicide ideation for heterosexual males. For sexual minority males, however, there appear no significant effects. Given the large coefficient for the effect of self-injury, we suspect that the differences in relationships are largely due to sample size differences, unlike the lack of effect on suicidal thoughts. The results for fighting present a similar story with a significant effect indicating increased risk for heterosexual males, but no statistically significant effect for sexual minority males despite higher effect sizes for one coefficient (suicide ideation for this predictor). Sexual abuse, conversely, indicates no significant effects for either male population.

**Table 2:** Self-injury and suicidal thoughts among males.

	Heterosexual Males				Sexual Minority Males			
	Self-Injury		Suicidal Thoughts		Self-Injury		Suicidal Thoughts	
	b	SE	b	SE	b	SE	b	SE
Bullied	.129	.032**	.102	.033**	.190	.102	.009	.103
Fighting	.093	.033**	.106	.033**	-.049	.103	.153	.106
Sexual Assault	.222	.165	.110	.165	.044	.328	-.130	.337
Substance Use	.163	.038**	.123	.037**	.395	.134**	.119	.136
Hardcore Drug Use	.040	.033	.023	.033	-.040	.089	-.030	.085
Sexual Behavior	-.143	.040**	-.058	.037	.149	.136	.026	.138
Sad/Hopeless	.956	.073**	1.150	.071**	.784	.251**	1.060	.244**
Fasting	.364	.118**	.246	.119*	.486	.326	.734	.338*
Diet Pills	-.049	.187	-.086	.187	.266	.464	-1.658	.578**
Purging	.099	.230	-.008	.237	-.051	.467	1.207	.556*
Constant	-1.871	.047	-1.852	.047	-1.380	.204	-1.138	.184
p			.472 (.044)			.822 (.090)		

* p < .05 ** p < .01

Observed effects from substance use indicate the substance use is positively related to self-injury and suicide ideation for heterosexual males, but only to self-injury for sexual minority males. Though, again this difference may be due to power differences between samples give the relatively similar coefficient size. Conversely, hardcore substance use appears to be unrelated to either behavior for either population. 

These results also show that sexual behavior is not observed to be related to suicide ideation, but it does appear to be related to self-injury. Specifically, among heterosexual males, sexual activity appears to significantly decrease the likelihood of engaging in self-injury. For sexual minority males, however, sexual activity appears to increase the propensity towards self-injury (albeit non-significantly). The equality test on these coefficients indicates that, though the individual effect does not appear significant for sexual minority males, the large difference between the two is significantly different, thus indicating that sexual activity has opposite effects depending on sexual orientation (p=.039). 

The measure of depression, feeling sad or hopeless, indicates a significant effect increasing likelihood of self-injury and suicide ideation for both orientations. Similarly, fasting among males also significantly increases the propensity towards these behaviors, though the effect on self-injury is not significant for sexual minority males. Other eating and dieting behaviors present a more specific story. Neither using diet pills nor purging are significant predictors of self-injury. Both indicators, however, significantly relate to suicide ideation, but only for sexual minority males. For diet pills, the relationship indicates that those using the pills are less likely to have suicidal thoughts, while those fasting are more likely to think about suicide. In both cases, these results are only found for sexual minority males and significantly differ from the non-relationship found for heterosexual males (p= .010 and .044, respectively).

The results for females are presented in Table 3. For heterosexual females, having been the victim of bullying significantly and positively relates to both self-injury and suicide ideation. Among sexual minority females, however, this relationship is only found to be significant for suicidal thoughts. Fighting, conversely, is non-significant for all categories and outcomes.

**Table 3:** Self-injury and suicidal thoughts among females.

	Heterosexual Females				Sexual Minority Females			
	Self-Injury		Suicidal Thoughts		Self-Injury		Suicidal Thoughts	
	b	SE	b	SE	b	SE	b	SE
Bullied	.170	.042**	.121	.045**	.184	.095	.230	.095*
Fighting	-.070	.054	-.028	.053	-.142	.084	-.004	.086
Sexual Assault	.429	.092**	.486	.092**	.402	.180*	.231	.185
Substance Use	.188	.035**	.110	.036**	.074	.081	.053	.082
Hardcore Drug Use	.154	.054**	.124	.053*	.027	.068	-.074	.071
Sexual Behavior	-.202	.041**	-.041	.040	.041	.087	.095	.089
Sad/Hopeless	.698	.063**	.863	.065**	.858	.157**	.963	.165**
Fasting	.462	.082**	.264	.085**	.334	.180	.197	.182
Diet Pills	-.230	.143	-.061	.143	.262	.247	.064	.260
Purging	.655	.133**	.394	.138**	-.117	.265	.507	.273
Constant	-1.558	.046	-1.661	.048	-.893	.127	-1.136	.136
p			.509 (.035)			.495 (.082)		

* p < .05 ** p < .01

Being the victim of sexual assault also varies slightly among orientations. For heterosexual females, sexual abuse is a significant predictor of self-injury and suicidal thoughts. Though the relationship with self-injury is also found for sexual minority females, the same cannot be said for suicide ideation among this group. The lack of an effect on suicide ideation among sexual minority females departs from prior findings and may be worthy of further investigation. This difference, however, is not significant (p=.217).

Substance use and hardcore substance use indicate some variation by sexual orientation as well. Both are significant predictors for heterosexual females for both self-injury and suicide ideation. For sexual minority females, however, these data indicate no significant relationship, with the hardcore substance use actually indicating a negative (non-significant) direction with suicidality and significantly differing from heterosexual females (p = .025). This suggests that looking at gender and orientation combinations is important in understanding for whom these substances lead to increased risks. For sexual activity, as with the findings for males, the data suggest no relationship with suicide ideation and opposite effects on self-injury depending on the subject's orientation. As with males, sexual behavior decreased risk for self-injury among heterosexual females, but increased (non-significantly) the risk for sexual minority females. This difference is statistically significant (p = .012).

Consistent with findings for males, feeling sad or hopeless indicates an increased likelihood towards self-injury and suicidal thoughts. This relationship is found regardless of sexual orientation. Eating behaviors, however, show several differences by orientation. Fasting and purging are both significant and positively related to NSSI and suicide ideation for heterosexual females, but are non-significant for sexual minority females. This indicates that heterosexual females who fast or purge are at a higher risk for self-injury or suicide ideation. Sexual minority females, however, are neither more nor less likely to engage in these risk behaviors based on their dieting practices.For the relationship between purging and self-injury, the effect is significantly different by orientation (p = .009).

vi. The model was significant based on a chi-square test for significant improvement in the log likelihood as compared to the null model. This was true for all four gender and sexual orientation combinations used here.

## Discussion

Despite the increased awareness of NSSI and other related risk factors associated with sexual minority youth, few studies have addressed the social and interpersonal antecedents and correlates associated with these antisocial behaviors. Most notably has been the absence of gender specific analyses in the empirical literature. The current study has investigated the unique correlates associated with male and female sexual minority youth who engage in NSSI and suicidal ideation.

In general, there were vast differences in the prevalence rates related to both NSSI and suicidal ideation across gender and reported sexual orientation. Regarding NSSI, 7% of heterosexual males and14% of heterosexual females reported engaging in this risky behavior, compared to 33% of sexual minority males and 48% of sexual minority females. Similarly, 8% of heterosexual males and 13% of heterosexual females reported suicidal ideation, compared to 36% of sexual minority males and 42% of sexual minority females. This research tested bullying, fighting, sexual abuse, substance use, sexual activity, depression, and various eating/dieting disorders as predictors of self-injury and suicide ideation. Among the four possible gender and orientation combinations used here, each predictor was significant as a predictor in at least one model, and most were significant in multiple models. Specifically, bullying, fighting, sexual abuse, substance use, depression, and two of the three dieting behaviors indicated a positive relationship with these outcomes. Only diet pills indicated a significant negative relationship and only among one group. 

In comparison to previous literature, some of the findings are consistent with prior research. For example, the effects from bullying are consistent with prior findings looking at the effects of various forms of abuse.^22,24,27,40,41^ Similarly, the effects from sexual abuse are consistent with prior research linking sexual abuse to NSSI.^22,27,40-44^ However, there are also some inconsistencies with previous research, which demonstrate the value in examining gender and orientation. For example, the substance use effects for males, with which there is significance for more common substances but not for hardcore substances, is opposite to those described in one previous study,^45^ but does conform to the relationship found in a study similarly using gender-specific models.^27^ This indicates that research lacking gender-specificity might fail to detect such relationships.

In addition to many differences between models in whether something is significant, these results identified several statistically significant differences in the effects between sexual orientations. Previous literature on the link between sexual activity and these outcomes has been mixed. Some findings suggest a significant, negative link with self-injury but not suicide,^27^ while others find no relationship.^46^ In the present study, sexual activity has opposite effects based on sexual orientation. Thus, failing to include the interaction of orientation and sexual activity in a model could lead to erroneously null findings. Similarly, substance use also varies depending on gender and orientation. Unhealthy dieting practices especially highlight the importance of gender and orientation being examined simultaneously along with other risk behaviors, as their relationships are clearly contingent on the gender/orientation configuration (with sexual minority males and heterosexual females at higher risk).

Overall, these findings add to the existing literature of NSSI and suicidal ideation by specifically examining gender differences and risk factors among sexual minority youth. Sexual minority youth confront an array of issues, including emotional isolation, social rejection, depression, and lowered self-esteem and body image,^19,47^ and are associated with a variety of risk factors, including substance use and sexual risk behaviors.^15^ As such, intervention efforts designed specifically to address not only the risk factors associated with NSSI and suicidal ideation, but also the social and behavioral issues more likely to exist among sexual minority youth is key in reducing these self-destructive behaviors. Specifically, intervention programs designed to identify and assist youth cope with the stress and social stigma often associated with one's sexual minority status are vital, particularly those that understand that potential gender differences in these self-harming behaviors may exist. These programs can serve as a proactive measure in either preventing these risky behaviors from occurring or recognizing them before they escalate into self-harm or suicidal thoughts.Identifying those students that are depressed, being bullied, have a negative self-image, or who may be engaging in substance use can aid in the prevention of self-harming behaviors if the necessary support system is available.Similarly, improving the culture within schools and providing support groups for sexual minority students has been shown to reduce both victimization and suicide attempts.^48^

These findings are naturally not without some limitations. First, both self-injury and suicide ideation were captured using a single-item measure. Several predictors similarly were limited by the available related questions. As such, specific types of self-injury could not be studied, nor can one be certain that the measure captures the full range of possible self-injuring behaviors. Second, these data are cross-sectional and therefore unable to properly estimate causality due to limited ability to address time-order and non-spuriousness. Though large, representative samples are generally helpful in terms of establishing generalizability, the design is limited in terms of causality. Finally, though three years of data were merged to create the largest dataset used to examine this topic heretofore,^27^ the power available is still limited for sexual minority youth. The analyses included 340 sexual minority females and 161 sexual minority males, with 162 and 53 engaging in NSSI, and 142 and 58 in suicide ideation, respectively. This is still more statistical power than used in prior analyses with representative samples, yet future research would be greatly strengthened through the use of an even larger sample given the rarity of these behaviors and sexual minority youth.

Overall, the findings here suggest significant differences by sexual orientation in addition to the already established differences by gender. Whereas previous research has built upon earlier research by developing better measures, moving toward large community based samples^46,49^ and using gender-specific models,^27^ the present findings suggest a need to examine specific groups within these larger communities. Though this study focuses on sexual orientation, future research should also consider other character traits or groups that may result in differing correlates and predictors.
